# Differential expression analysis for individual cancer samples based on robust within-sample relative gene expression orderings across multiple profiling platforms

**DOI:** 10.18632/oncotarget.11996

**Published:** 2016-09-13

**Authors:** Qingzhou Guan, Rou Chen, Haidan Yan, Hao Cai, You Guo, Mengyao Li, Xiangyu Li, Mengsha Tong, Lu Ao, Hongdong Li, Guini Hong, Zheng Guo

**Affiliations:** ^1^ Key Laboratory of Ministry of Education for Gastrointestinal Cancer, Department of Bioinformatics, Fujian Medical University, Fuzhou, 350001, China; ^2^ Department of Preventive Medicine, School of Basic Medicine Sciences, Gannan Medical University, Ganzhou, 341000, China

**Keywords:** gene expression profiling, multiple platforms, differentially expressed genes, heterogeneity of cancer, individual level

## Abstract

The highly stable within-sample relative expression orderings (REOs) of gene pairs in a particular type of human normal tissue are widely reversed in the cancer condition. Based on this finding, we have recently proposed an algorithm named *RankComp* to detect differentially expressed genes (DEGs) for individual disease samples measured by a particular platform. In this paper, with 461 normal lung tissue samples separately measured by four commonly used platforms, we demonstrated that tens of millions of gene pairs with significantly stable REOs in normal lung tissue can be consistently detected in samples measured by different platforms. However, about 20% of stable REOs commonly detected by two different platforms (e.g., Affymetrix and Illumina platforms) showed inconsistent REO patterns due to the differences in probe design principles. Based on the significantly stable REOs (FDR<0.01) for normal lung tissue consistently detected by the four platforms, which tended to have large rank differences, *RankComp* detected averagely 1184, 1335 and 1116 DEGs per sample with averagely 96.51%, 95.95% and 94.78% precisions in three evaluation datasets with 25, 57 and 58 paired lung cancer and normal samples, respectively. Individualized pathway analysis revealed some common and subtype-specific functional mechanisms of lung cancer. Similar results were observed for colorectal cancer. In conclusion, based on the cross-platform significantly stable REOs for a particular normal tissue, differentially expressed genes and pathways in any disease sample measured by any of the platforms can be readily and accurately detected, which could be further exploited for dissecting the heterogeneity of cancer.

## INTRODUCTION

Recently, we have reported an interesting biological phenomenon that the within-sample relative expression orderings (REOs) of gene pairs in a particular type of normal tissue are highly stable but widely reversed in the corresponding cancer tissue. Based on this finding, we have developed an algorithm, named *RankComp* [1], to identify differentially expressed genes (DEGs) and deregulated pathways in each disease tissue in comparison with its own previously normal state by exploiting the reversal REO patterns of this disease sample [1]. Totally different from the traditional population-level case-control comparison methods, such as T-Test [2], SAM [3], Limma [4] and RanProd [5], the individual-level analysis can capture the heterogeneity of cancer and help us study cancer subtype-specific mechanisms and develop cancer prognostic biomarkers [6]. Especially, *RankComp* is an economic and efficient method which can identify DEGs for individual disease samples measured by different laboratories by fully using previously accumulated gene expression data of normal samples [1].

It is well known that gene expression profiling is susceptible to various technical artifacts or ‘batch effects’ introduced by differences in laboratory conditions, reagent lots and personals [7–13], especially when studies have to be carried out over a long period of time or when clinical specimens originate from different hospitals [9]. Current batch effect adjustment or data normalization algorithms, such as DWD [8], XPN [14], PAMR [15], SVA [16] and EB [17], are usually inadequate and may even distort the real biological signals [18, 19]. In contrast, because the within-sample REOs of gene pairs are insensitive to experimental batch effects [20–22], the application of *RankComp* obviates the need of batch effect adjustment and inter-sample normalization. As validated in our previous studies, the within-sample REOs are highly reproducible and comparable between data produced by different laboratories using the same or similar platforms [1, 6]. However, the within-sample REOs may be subject to a certain degree of uncertainty in samples measured by different platforms due to the differences in probe design principles. Thus, it is necessary to further evaluate the cross-platform properties of within-sample REOs in order to extend the application scope of the individual-level differential expression analysis. Another problem of the current *RankComp* algorithm is that it is based on REOs that are highly stable in a pre-defined percentage (e.g., 99%) of normal samples, which is lack of statistical control and may limit the detection power of DEGs in individual samples. Thus, it is also necessary to evaluate the performance of *RankComp* when using significantly stable gene pairs, selected with statistical control rather than a pre-defined percentage, in a particular type of normal tissue as the basis for the individual-level differential expression analysis.

In this article, we firstly compared gene expression profiles generated with four commonly used gene expression profiling platforms (Affymetrix, Illumina, Agilent microarray platforms and a RNA-sequencing platform) for normal lung and colorectal tissues, respectively. For each type of normal tissue, we showed that tens of millions of gene pairs with significantly stable REOs, especially those with large expression differences, can be consistently detected in samples measured by different platforms. Then, we showed that, comparing with *RankComp* based on gene pairs with highly stable REOs in at least 99% normal samples, *RankComp* based on significantly stable REOs in normal samples can detect much more DEGs for each disease sample with slightly decrease of precision. Finally, based on the individual-level differential expression analysis, we applied the individual-level pathway analysis to reveal some common and subtype-specific functional mechanisms of lung adenocarcinoma and colon adenocarcinoma, respectively.

## RESULTS

### Significantly stable REOs in normal samples measured by four platforms

**Figure 1:**
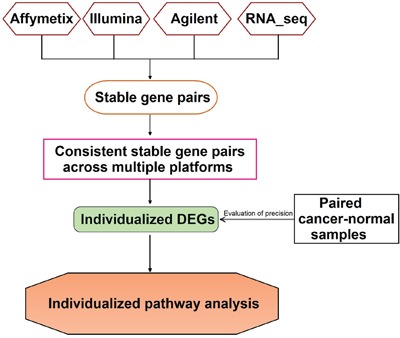
The flowchart of the analysis procedure

For a particular type of normal tissue, we focused on evaluating the consistency between the within-sample relative expression orderings (REOs) in samples separately measured by four platforms, including three commonly used microarray platforms (Affymetrix, Illumina, Agilent) and a RNA-sequencing platform. All the data used in this study are described in Table 1 and the flowchart of the analysis procedure is described in Figure 1.

**Table 1:** Description of normal sample data and paired cancer-normal sample data used in this study

	GEO Acc or Data source	Platform	Normal sample size^a^	Tumor sample size
The normal sample data for REO evaluation				
		**Lung**		
SetA	GSE19804	Affymetrix GPL570	60	
	GSE18842	Affymetrix GPL570	45	
	GSE27262	Affymetrix GPL570	25	
	GSE31210	Affymetrix GPL570	20	
SetB	GSE19188	Affymetrix GPL570	65	
SetA	GSE32863	Illumina GPL6884	58	
SetB	GSE31267	Illumina GPL6947	24	
SetA	GSE40588	Agilent GPL6480	60	
SetB	GSE15197	Agilent GPL6480	13	
	GSE57148^b^	Illumina GPL11154	91	
		**Colorectal**		
SetA	GSE21510	Affymetrix GPL570	25	
	GSE18105	Affymetrix GPL570	17	
	GSE4107	Affymetrix GPL570	10	
SetB	GSE8671	Affymetrix GPL570	32	
SetA	GSE56789	Illumina GPL10558	40	
SetB	GSE31279	Illumina GPL6104	32	
	GSE43841	Illumina GPL14951	6	
SetA	GSE46271	Agilent GPL14550	22	
	GSE50114	Agilent GPL6480	9	
SetB	GSE28000	Agilent GPL4133	23	
	GSE50760^b^	Illumina GPL11154	18	
The paired cancer-normal sample data for the performance of *RankComp* evaluation				
		**Lung**		
	GSE27262	Affymetrix GPL570	25	25
	GSE32863	Illumina GPL6884	57	57
	TCGA_luad^b^	IlluminaHiSeq_RNASeqV2	58	58
		**Colorectal**		
	GSE8671	Affymetrix GPL570	32	32
	GSE31279	Illumina GPL6104	32	32
	TCGA_coad^b^	IlluminaHiSeq_RNASeqV2	26	26

Note:

a To determine stable gene pairs for a particular type of normal tissue, only the normal sample sizes were described for the datasets.

b Denotes mRNA_seq data, especially TCGA_luad and TCGA_coad denote paired lung adenocarcinoma and colon adenocarcinoma samples from TCGA, respectively.

Firstly, for the Affymetrix platform, we collected a set of 150 normal lung tissue samples from four datasets (GSE19804, GSE18842, GSE27262 and GSE31210) and another set of 65 normal lung tissue samples from the GSE19188 dataset, referred to as SetA and SetB, respectively. From SetA and SetB, 197,546,446 and 195,767,556 significantly stable REOs (binomial test, FDR< 0.01) were identified, respectively. The two lists of significantly stable REOs had 190,118,028 overlaps, among which 98% showed the same REO patterns in SetA and SetB, indicating that the significantly stable REOs of gene pairs were highly reproducible (binomial test, *p*<1.0-16). As shown in Table 2, 94.34% of the significantly stable REOs for SetA with 150 samples could be found in SetB with 65 samples, and 95.19% of the significantly stable REOs for SetB could be found in SetA. To further evaluate the sample size required for detecting significantly stable REOs, we resettled the GSE31210 dataset with 20 samples as SetB' and all the other 195 samples as SetA' and found that 88.50% of the significantly stable REOs found in SetA' could be found in SetB'. Similar results were observed in the normal lung tissue samples measured by the Illumina and Agilent platforms, respectively, as described in Table 2. Especially, for the data measured by the Agilent platform, 89.06% of the significantly stable REOs found in SetA with 58 samples could be found in SetB with 24 samples. For the data measured by the Illumina platform, 78.33% of the significantly stable REOs detected from SetA with 60 samples could be found in SetB with only 13 samples. Similar results were observed for the normal colorectal tissue, as described in Table 2.

**Table 2:** Reproducibility of significantly stable REOs in normal samples measured by each of the platforms

	Label	Normal sample size	Gene^#^	Number of stable REOs	Number of overlaps	POG_12_	POG_21_	Consistency	P
**Lung**									
Affymetrix	SetA	150	20283	197,546,446	190,118,028	0.9434	0.9519	0.9802	<1.0-16
	SetB	65		195,767,556					
Illumina	SetA	58	23364	251,964,302	231,498,834	0.8906	0.9061	0.9694	<1.0-16
	SetB	24		247,667,868					
Agilent	SetA	60	19596	181,534,752	151,185,241	0.7833	0.9105	0.9406	<1.0-16
	SetB	13		156,176,364					
**Colorectal**									
Affymetrix	SetA	52	20283	193,475,574	184,134,774	0.9136	0.9135	0.96	<1.0-16
	SetB	32		193,501,698					
Illumina	SetA	40	17789	148,902,375	131,019,285	0.8048	0.8723	0.9147	<1.0-16
	SetB	38		137,385,589					
Agilent	SetA	31	18583	145,935,881	121,390,845	0.8099	0.87	0.9736	<1.0-16
	SetB	23		135,855,195					

Note:

# denotes the number of genes of SetA and setB measured by a particular platform. POG12 (or POG21) denotes the percentage of the significantly stable gene pairs (FDR<0.01) detected from SetA (or SetB) that are consistently detected in SetB (or SetA). Consistency denotes the percentage of overlapped gene pairs that display the same REO patterns between SetA and SetB and P denotes the significance of the consistency.

**Figure 2:**
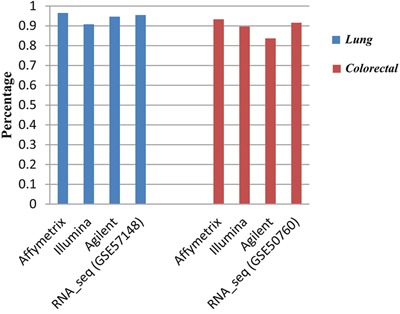
The percentage of the gene pairs with significantly stable REOs (FDR<0.01) in all measured gene pairs

Notably, for both the normal lung and colorectal tissue, the gene pairs with significantly stable REOs found in each dataset involved all genes measured by the corresponding platform. For each type of tissue, when combining all the samples measured by a platform, above 80% of all the possible gene pairs were significantly stable (FDR<0.01), as shown in Figure 2. In addition, above 80% of the significantly stable REOs detected in the combined data measured by a platform could be found with a relatively small sample size (about 20 samples), as described in Supplementary Table S1.

The above results together indicated that the REOs of gene pairs are widely stable in a particular type of human normal tissue and most of them could be found with only about 20 samples.

### Cross-platform significantly stable REOs in normal tissue samples

Then, we evaluated the consistency between the significantly stable REOs detected by different platforms. For each of the three microarray platforms, we defined the REOs consistently detected in SetA and SetB measured by the platform for a type of normal tissue as the stable REOs of the platform for this type of normal tissue. Especially, we only analyzed the gene pairs consisting of genes commonly detected by all the four platforms.

For the 94,145,902 significantly stable REOs detected from the normal lung tissue samples measured by the Affymetrix platform, 85.50% were also detected from the data measured by the Illumina platform, among which 82.37% showed the same REO patterns in the samples measured by the two platforms (binomial test, *p*<1.0-16). For the 66,305,728 significantly stable REOs consistently detected by the above two platforms, 79.91% were included in the 77,825,426 significantly stable REOs found in the data measured by the Agilent platform and the consistency increased to 92.1%. Furthermore, for the 48,802,858 significantly stable REOs consistently detected by the three microarray platforms, 98.01% were included in the 99,202,212 significantly stable REOs (binomial test, FDR<0.01) detected by the RNA-sequencing platform and the consistency further increased to 96.79%. Totally 46,295,854 gene pairs with significantly stable REOs (FDR<0.01) were consistently detected by the four platforms for the normal lung samples. Similar results were also observed for normal colorectal tissue, as described in Table 3.

**Table 3:** Cross-platform evaluation of the significantly REOs for normal tissues

	Number of stable REOs	Number of overlaps	POG_12_	POG_21_	Consistency	P
**lung**						
Affymetrix	94,145,902	80,493,915	0.7043	0.7471	0.8237	<1.0-16
Illumina	88,746,864					
Affy_Illu	66,305,728	52,986,997	0.736	0.6271	0.921	<1.0-16
Agilent	77,825,426					
Affy_Illu_Agi	48,802,858	47,832,844	0.9486	0.4667	0.9679	<1.0-16
RNA_seq (GSE57148)	99,202,212					
**Colorectal**						
Affymetrix	100,855,012	78,495,790	0.6729	0.7569	0.8645	<1.0-16
Illumina	89,653,488					
Affy_Illu	67,862,351	52,201,960	0.7347	0.6223	0.9551	<1.0-16
Agilent	80,116,625					
Affy_Illu_Agi	49,856,959	48,851,749	0.9662	0.4453	0.9861	<1.0-16
RNA_seq (GSE50670)	108,187,244					

Note: Affy_Illu denotes stable gene pairs consistently detected from the data measured by Affymetrix and Illumina platforms. Similarly, Affy_Illu_Agi denotes stable gene pairs consistently detected from the data measured by Affymetrix, Illumina and Agilent platforms.

The above results indicated that the cross-platform ability of significantly stable REOs increases as the significantly stable REOs can be consistently detected in increasing number of platforms. A possible explanation could be that the REOs kept across multiple platforms with different probe design principles tend to have large rank differences which are difficult to be reversed by the probe detection biases of various platforms. To illustrate this possibility, for the significantly stable REOs commonly detected by the Affymetrix and Illumina platforms for normal lung tissue, we compared the rank differences between the gene pairs with consistent REOs and the gene pairs with inconsistent REOs detected by the two platforms using the GSE19188 dataset. As expected, the median of the rank differences of gene pairs with consistent REOs was 6855, which was significantly larger than that (median=3144) of the gene pairs with inconsistent REOs (Wilcoxon rank sum test, *p*<1.0-16).

In summary, for a particular type of human normal tissue, significantly stable within-sample REOs especially for gene pairs with large expression differences are largely consistent across samples measured by different platforms.

### Individualized DEGs detection based on cross-platform significantly stable REOs

As described above, totally 46,295,854 gene pairs with significantly stable REOs (FDR<0.01) were consistently detected in the normal lung samples separately measured by the four platforms. Based on these significantly stable REOs, we applied *RankComp* [1] to detect differentially expressed genes (DEGs) in a given cancer sample compared with its own previously (usually unknown) normal state. The detail of the *RankComp* algorithm was described in [1] and briefly in the METHODS section. We evaluated the performance of *RankComp* using cancer samples with paired adjacent normal samples, assuming that the previously normal state of a cancer tissue could be approximately represented by the adjacent normal tissue of the cancer tissue. After identifying DEGs for each cancer sample, we evaluated the precision of DEGs identified for this cancer sample using the observed expression differences (up- or down-regulations) between the cancer sample and the paired adjacent normal sample as the benchmark (see Methods). Considering that other individual-specific factors irrelevant to the cancer condition may induce transcriptional alternations in the cancer sample, we focused on individualizing the population-level DEGs predetermined to ensure the DEGs identified in individual cancer samples to be associated with cancer. We also evaluated the performance of *RankComp* for individualized differential expression analysis based on the highly stable REOs (stable in at least 99% of the samples) consistently detected in the normal lung and colorectal tissue samples measured by the four platforms, respectively.

**Figure 3:**
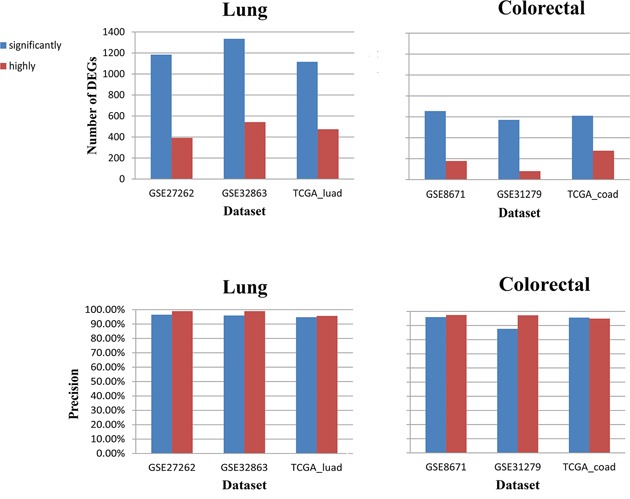
*RankComp* based on significantly stable REOs can detect much more DEGs with slightly decreased precision for each disease sample than *RankComp* based on highly stable REOs (stable in above 99% samples)

For lung cancer, using the GSE19188 and GSE19804 datasets, we firstly detected 12,359 and 8,681 DEGs (Student's t-test, FDR<0.01) between the cancer and normal tissues, respectively. The two lists of DEGs had 6,929 overlaps, among which 98.46% showed the same deregulation directions in the cancer tissues in the two datasets (binomial test, *p*<1.0-16). We defined the 6,822 reproducible DEGs as the population-level DEGs for lung cancer. Then, using three datasets with paired lung cancer and adjacent normal samples separately measured by three different platforms, we evaluated the performance of *RankComp* in individualizing the population-level DEGs. For each cancer sample, we performed *RankComp* based on the 46,295,854 gene pairs with significantly stable REOs (FDR<0.01) consistently detected in the normal lung samples measured by the four platforms. For the 25 lung cancer samples of the GSE27262 dataset measured by the Affymetrix platform, *RankComp* identified averagely 1,184 DEGs per sample with averagely 96.51% precision according to the observed expression differences between the cancer samples and their paired adjacent normal samples. We also evaluated *RankComp* using the GSE32863 and TCGA-luad (lung adenocarcinoma samples from TCGA) datasets which included 57 and 58 paired cancer and adjacent normal samples measured by the Illumina microarray platform and the Illumina HiSeq 2000 platform, respectively. Averagely 1,335 and 1,116 DEGs were identified per cancer sample and the average precisions were 95.95% and 94.78% for the two datasets, respectively. In contrast, based on the 21,789,916 highly stable REOs (stable in at least 99% of the samples) consistently detected in the normal lung samples measured by the four platforms, the average precision increased to 98.96%, 98.97 % and 95.65% but averagely only 392, 542 and 474 DEGs were identified per sample in the three datasets, respectively. Similar results were also observed for colorectal cancer, as shown in Figure 3.

The above results showed that *RankComp* based on significantly stable REOs exhibited greatly enhanced detection power at the cost of slightly decrease of precision, compared with *RankComp* based on highly stable REOs.

### Individualized pathway analysis based on individualized DEGs

After identifying DEGs for a disease sample, we can detect deregulated pathways for this disease sample. Here, we analyzed all the 515 lung adenocarcinoma samples and the 285 colon adenocarcinoma samples documented in TCGA to illustrate this application.

**Figure 4:**
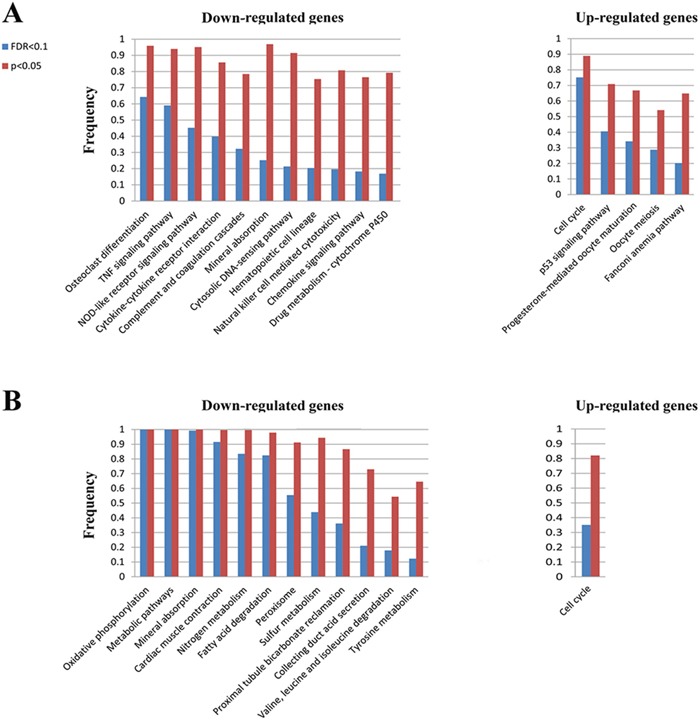
The KEGG pathways separately enriched with up- and down-regulated genes in at least 10% of the TCGA lung adenocarcinoma samples A. and the TCGA colon adenocarcinoma samples B

First, we detected pathways separately enriched with up- or down-regulated genes for each of the 515 lung adenocarcinoma samples (hypergeometric test, FDR<0.1) [23]. As shown in Figure 4A, some well-known cancer pathways could be commonly altered in lung adenocarcinoma samples. For examples, the ‘Osteoclast differentiation’ [24] and ‘TNF signaling’ [25] significantly enriched with down-regulated genes in about 60% of the 515 samples (FDR<0.1) and the coverage increased to above 90% with a looser significance threshold of *p*<0.05. In addition, the ‘Cell cycle’ [26] pathway significantly enriched with up-regulated genes in about 75% or 90% (with FDR<0.1 or *p*<0.05) of the 515 samples. In contrast, some pathways could be subtype-specific. For example, the ‘Fanconi anemia pathway’ significantly enriched with up-regulated genes in about 20% or 65% (with FDR<0.1 or *p*<0.05) of the 515 samples, indicating that it might be associated with cancer prognosis [27]. For another example, the ‘Chemokine signaling pathway’ significantly enriched with down-regulated genes in about 18% or 77% (with FDR<0.1 or *p*<0.05) of the 515 lung cancer samples, indicating that it might also be associated with cancer prognosis [28].

Similarly, we performed pathway enrichment analysis for each of the 285 colon adenocarcinoma samples. As shown in Figure 4B, two pathways (‘oxidative phosphorylation’ and ‘metabolic pathway’) were significant in all the 285 samples with FDR<0.1 and another five pathways (‘Mineral absorption’, ‘Cardiac muscle contraction’, ‘Fatty acid degradation’, ‘Nitrogen metabolism’ and ‘Peroxisome’) were also significant in above 90% of the 285 samples when defined with a looser significance threshold (*p*<0.05). Thus, these pathways, such as the ‘metabolic’ [29] and ‘oxidative phosphorylation’ [30] pathways, could be commonly altered in colon cancer. In contrast, some other pathways such as ‘valine, leucine and isoleucine degradation’ [31] and ‘Tyrosine metabolism’ [32] pathways could be subtype-specific and thus could be associated with cancer prognosis. Especially, with p<0.05, the ‘Cell cycle’ pathway significantly enriched with up-regulated genes in 89% of the 515 lung adenocarcinoma and in 82% of the 285 colon adenocarcinoma samples, indicating that this pathway might be commonly deregulated in cancer [33]. In addition, the ‘Mineral absorption’ pathway significantly enriched with down-regulated genes in 97% of the 515 lung adenocarcinoma and in 100% of the 285 colon adenocarcinoma samples, indicating that this pathway might also be commonly deregulated in cancer [34, 35].

The above results suggested that individualized pathway analysis could provide hints for revealing common and subtype-specific functional mechanisms of cancer. The functional analysis results also provided extra evidence for the authenticity of individualized DEGs at the functional level.

## DISCUSSION

As demonstrated in this article, tens of millions of gene pairs with significantly stable REOs in a particular type of normal tissue, especially those with large expression differences, can be consistently detected by different platforms. This provides the basis for individual-level differential expression analysis for cancer samples measured by different platforms. Compared with *RankComp* based on highly stable REOs (e.g., stable in above 99% samples), *RankComp* based on significantly stable REOs can detect much more DEGs with slightly decrease of precision for each disease sample, as demonstrated by the results for both lung cancer and colorectal cancer. Individual-level DEGs analysis naturally enables us to perform pathway analysis at the individual level, which could reveal common functional mechanisms as well as subtype-specific functional mechanisms of cancer. This is totally different from the traditional population-level pathway analysis which cannot discriminate whether a significant pathway is altered in a group of patients (i.e., a subtype) or all patients. Furthermore, our results showed that almost all gene pairs had significantly stable REOs across samples for a given normal tissue. This indicated that the relative ordering of gene expression is overall stable in a particular type of normal human tissue, indicating that genes may need to express in a comprehensive coordination structure to carry normal function systematically [1].

Based on the significantly stable REOs consistently detected by multiple platforms for a particular type of tissue, DEGs and deregulated pathways for any disease sample measured by any of these platforms can be readily detected. This could be of particular valuable when we need to analyze multiple datasets of disease samples measured by different platforms to identify and validate various cancer signatures (such as prognostic signatures) [36]. Moreover, for a particular normal tissue, our result showed that, the significantly stable REOs consistently detected in more platforms tend to be more likely to remain consistent in a new platform. Especially, almost all (above 97%) significantly stable REOs consistently detected (binomial test, FDR<0.01) by the three microarray platforms could be reproducibly found by the RNA-sequencing platform. This might be helpful for analyzing disease samples measured by a less commonly used platforms when no or insufficient normal samples are measured by the platform for determining the stable REOs for this platform. Notably, a major limitation of selecting cross–platform stable REOs is that many truly stable REOs could be lost. As shown in Table 3, about half of the stable REOs detected by a particular platform will be lost when screening the stable REOs from samples measured by the four platforms for lung and colorectal tissue. Although our results indicated that it might be sufficient to detect DEGs based on the cross-platform stable REOs, the effects of using a certain percentage of stable REOs on the DEGs detection power need to be further studied.

In summary, a large fraction of the widely stable REOs in a particular type of normal tissue can be consistently detected in samples measured by different platforms. By fully using previously accumulated gene expression data of normal samples, *RankComp* is an economic and efficient method which can identify DEGs for individual disease samples measured by different platforms. Moreover, the individual-level analysis of DEGs can also provide the possibility to identify robust diagnostic and prognostic biomarker for precision medicine [36].

## MATERIALS AND METHODS

### Data and preprocessing

The gene expression profiles analyzed in this study are described in Table 1. The data generated with three commonly used microarray platforms (Affymetrix, Illumina, Agilent) were downloaded from Gene Expression Omnibus [37] (GEO, http://www.ncbi.nlm.nih.gov/geo/) and the mRNA-seq data measured by RNA-sequencing platform were downloaded from GEO and TCGA [38] (http://cancergenome.nih.gov/).

For the data measured by the Affymetrix platform, we downloaded the raw mRNA expression data (.CEL files) and used the Robust Multi-array Average algorithm for background adjustment [39]. For the data measured by the Illumina platform, we directly downloaded the processed data. For the data measured by the Agilent platform, we downloaded the raw fluorescent signal intensities data of the channel (gMedianSignal or rMedianSignal) for normal samples and used the intensities to minus the corresponding background signal intensities as the probe-expression matrix. Especially, for the data of TCGA, we directly downloaded the expression data of level 3.

For array-based data, each probeset ID was mapped to Entrez gene ID with the corresponding platform file. If a probeset was mapped to multiple or zero gene, then the data of this probeset was deleted. If multiple probesets were mapped to the same gene, the expression value for the gene was defined as the arithmetic mean of the value of multiple probesets. For the sequence-based data form GEO, we directly downloaded the processed data and each Gene Symbol was mapped to Entrez gene ID with biological DataBase network (bioDBnet). For the sequence-based data of level 3 from TCGA, we removed genes whose expression measurements were at or below a noise threshold of 0.2 normalized counts in at least 75% of samples [40].

### Identification of significantly stable REOs in normal tissue

The REO of two genes, A and B, is denoted as A >B (or B<A) if gene A has a higher (or lower) expression level than gene B. The significance of a REO is determined by a binomial test [41] as follows:

(1) $$p=1-\overset{k-1}{\underset{i=0}{\sum^{\text{​}}}}\left(\begin{array}{c} n\\ i \end{array}\right)p_{0}^{i}{\left(1-p_{0}\right)}^{n-i}$$

where *n* denotes the total number of normal samples, *k* denotes the number of samples that have a certain REO pattern (e.g., A>B or A<B) in n normal samples and *p_0_* (*p_0_*=0.5) is the probability of observing a certain REO pattern in a normal sample by chance. Then, the *p*-values were adjusted using the Benjamini and Hochberg method [42].

### Evaluation of the reproducibility of the significantly stable REOs

We used the POG (Percentage of Overlapping Gene pairs) score [43, 44] to evaluate the reproducibility of significantly stable gene pairs identified from two independent datasets. If two lists of stable gene pairs, list 1 with length *L_1_* and list 2 with length *L_2_*, have *n* overlaps, among which *k* have the same REO patterns, then the POG score from list 1 (or list 2) to list 2 (or list 1), denoted as POG_12_ (or POG_21_), is calculated as *k/L_1_* (or *k/L_2_*), and the concordance score is calculated as *k/n*. The probability of observing the concordance score by chance is calculated with the binomial distribution model as described above, where *p_0_* (*p_0_* =0.5) is the probability of a gene pair having the same REO patterns in the two lists by chance.

### Performance evaluation of *RankComp*

The detail of the *RankComp* algorithm is described in [1]. Briefly, for each cancer sample, gene pairs with reversal ordering in comparison with their stable ordering in normal samples are firstly determined as reversal gene pairs by *RankComp*. Then, to determine whether a given gene A is differentially expressed in a given disease sample, Fisher's exact test [45] is used to test the null hypothesis that the proportion of reversal gene pairs supporting the up-regulation of gene A is equal to the proportion of gene pairs supporting its down-regulation. For a given gene A, if its ordering is significantly lower (or higher) than that of another gene in normal samples but this REO is reversed in a cancer sample, then this reversal gene pair could support up-regulation (or down-regulation) of gene A in the cancer sample. If gene A itself is not changed in expression level, the effect of the expression changes of other genes on the upward or downward shift in the rank of gene A is assumed to be a random event. Finally, a filtering process is utilized to retain only those DEGs which are still significant with Fisher's exact test after excluding their coupled gene pairs including any other DEGs.

We used paired cancer and adjacent normal samples to evaluate the performance of *RankComp*, assuming that the unknown previously normal state of a cancer tissue could be approximately represented by the adjacent normal tissue of the cancer tissue. After identifying DEGs for one cancer sample, if the deregulation directions (up- or down-regulations) of DEGs are consistent with the deregulation directions observed in the cancer sample compared with its own adjacent normal sample, then they are defined as true positives (TP); otherwise, false positives (FP). The precision rate is calculated as TP/(TP+FP) for each cancer sample. To ensure the association between the individualized DEGs and cancer, we restricted our evaluation to the reproducible population-level DEGs predetermined using two datasets for each type of cancer. For each cancer, the statistical significance of the concordance score between two lists of DEGs between cancer samples and normal controls, detected by Student's t-test, is calculated by the binomial distribution model as described above. Finally, the DEGs reproducibly detected in the two datasets for each cancer are defined as the population-level DEGs associated with the cancer.

### The KEGG pathways

Data of 223 pathways covering 6290 unique genes were extracted from the Kyoto Encyclopedia of Genes and Genomes (KEGG) [46] on 10 May 2015. The hypergeometric distribution model is used to determine the significance of biological pathways enriched with up- and down-regulated DEGs, respectively [23]. The *p*-values are adjusted using the Benjamini and Hochberg procedure.

## SUPPLEMENTARY MATERIALS TABLE



## Abbreviations

REOs: relative expression orderings
DEGs: differentially expressed genes
GEO: Gene Expression Omnibus
KEGG: Kyoto Encyclopedia of Genes and Genomes
POG: Percentage of Overlapping Gene pair
FDR: False Discovery Rate
TP: true positives
FP: false positives
TCGA_luad: lung adenocarcinoma samples from TCGA
TCGA_coad: colon adenocarcinoma samples from TCGA.
