# Supplementary material for: Differential expression analysis for individual cancer samples based on robust within-sample relative gene expression orderings across multiple profiling platforms
Source: Oncotarget. 2016 Sep 13;7(42):68909–20. doi: 10.18632/oncotarget.11996 (PMC5356599; doi:10.18632/oncotarget.11996)
Supplement: Supplementary file 1 [file oncotarget-07-68909-s001.pdf]

Differential expression analysis for individual cancer samples based on robust within-sample relative gene expression orderings across multiple profiling platforms

SUPPLEMENTARY TABLE

Supplementary Table S1: Reproducibility evaluation of significantly stable REOs detected in small samples for each platform

	Platform	Normal	Number	POG
		**Lung**		
GSE31210	Affymetrix	20	191,721,331	0.8923
GSE31267	Illumina	24	247,667,868	0.9168
GSE15197	Agilent	13	156,176,364	0.7923
		**Colorectal**		
GSE21510	Affymetrix	25	190,854,700	0.9475
GSE31279	Illumina	32	135,225,886	0.8578
GSE46271	Agilent	22	137,856,117	0.8453

Note: POG denotes the percentage of the significantly stable REOs for the combined data for each platform are included in the corresponding small sample dataset.
